# Characterization of the Protective HIV-1 CTL Epitopes and the Corresponding HLA Class I Alleles: A Step towards Designing CTL Based HIV-1 Vaccine

**DOI:** 10.1155/2014/321974

**Published:** 2014-03-18

**Authors:** Sajib Chakraborty, Taibur Rahman, Rajib Chakravorty

**Affiliations:** ^1^Department of Biochemistry and Molecular Biology, Faculty of Biological Sciences, University of Dhaka, Dhaka 1000, Bangladesh; ^2^Department of EEE, University of Melbourne, National ICT Australia, Melbourne, VIC 3010, Australia

## Abstract

Human immunodeficiency virus (HIV) possesses a major threat to the human life largely due to the unavailability of an efficacious vaccine and poor access to the antiretroviral drugs against this deadly virus. High mutation rate in the viral genome underlying the antigenic variability of the viral proteome is the major hindrance as far as the antibody based vaccine development is concerned. Although the exact mechanism by which CTL epitopes and the restricting HLA alleles mediate their action towards slow disease progression is still not clear, the important CTL restricted epitopes for controlling viral infections can be utilized in future vaccine design. This study was designed for the characterization the HIV-1 optimal CTL epitopes and their corresponding HLA alleles. CTL epitope cluster distribution analysis revealed only two HIV-1 proteins, namely, Nef and Gag, which have significant cluster forming capacity. We have found the role of specific HLA supertypes such as HLA B∗07, HLA B∗58, and HLA A∗03 in selecting the hydrophobic and conserved amino acid positions within Nef and Gag proteins, to be presented as epitopes. The analyses revealed that the clusters of optimal epitopes for Nef and p24 proteins of HIV-1 could potentially serve as a source of vaccine.

## 1. Introduction

Human immunodeficiency virus (HIV), a retrovirus that belongs to the Lentiviridae family, is the causative agent of acquired immunodeficiency syndrome (AIDS). HIV genome is composed of 9.8 Kb positive-sense, single-stranded RNA which is reverse transcribed by the enzyme reverse transcriptase to viral DNA upon its entry into the host cell [1]. Between the two types of HIV (HIV-1 and HIV-2), HIV-1 is more virulent and responsible for most of the HIV infections globally. Human immunodeficiency virus-1 (HIV-1) has infected more than 60 million people and caused nearly 30 million deaths worldwide [2]. In Asia, an estimated 4.9 million people were living with HIV in 2009, about the same as 5 years earlier. Most national HIV epidemics appear to have stabilized. Incidence fell by more than 25% in India, Nepal, and Thailand between 2001 and 2009. The epidemic remained stable in Malaysia and Sri Lanka during this time period. Incidence increased by 25% in Bangladesh and Philippines between 2001 and 2009 even as the countries continue to have relatively low epidemic levels [3]. Although the antiretroviral therapy has proven to be effective in controlling the infection in the developed world, only one-fourth populations in the developing world can afford these medications due to less accessibility. Consequently vast majority of people are living with a constant threat of HIV infection and death by AIDS. In this devastating situation of world AIDS epidemic, there is an urgent need of developing effective HIV vaccine as no vaccine is proved to be efficacious to control HIV infection. To combat this deadly virus, its genome, proteome, pathogenesis, and mechanisms of evasion of immune response should be studied in great detail.

HIV possesses complex RNA genome and contains nine genes which can be classified into 3 functional groups. Among these genes, Gag, Pol, and Env are structural genes, Tat and Rev are regulatory genes, whereas the rest of the genes (Vpu, Vpr, Vif, and Nef) fall into the accessory category of genes [4]. Early HIV replication cycle begins with the recognition of the target cells (mainly CD4^+^ T cells) by the mature virion and continues as virion core particles enters and facilitates its integration to the genomic DNA of the chromosome of the host cell. The late phase begins with the regulated expression of the integrated proviral genes and ends up with virus budding and maturation.

Gag gene encodes for 3 proteins: matrix (p17), capsid (p24), and nucleocapsid (p7) which are translated as polyproteins and later undergo a cleavage at specific site to give rise to three individual proteins. Pol gene also encodes for a polyprotein which has similar fate like Gag-poly-protein as it is also cleaved by viral protease into three different proteins: reverse transcriptase, protease, and integrase, whereas the Env gene encodes for a single glycoprotein (gp160) which later is cleaved into two proteins: surface glycoprotein gp120 and transmembrane protein gp41. Besides these some other regulatory proteins are also included in the HIV proteome such as Nef, Vpr, Tat, and Ref. In Table 1, we have collected functions of HIV proteins from Uniprot database of HIV, http://www.uniprot.org/uniprot/P04585.

High antigenic variability that results from the high mutation rate can be considered as the characteristic features of retrovirus such as HIV. This vast genetic heterogeneity of HIV not only helps the virus to evade selective pressures exerted by immune response and drug but also facilitates the viral evolution in a faster speed [5]. Phylogenetic studies showed the presence of three distinct groups: M (Major), O (Outliers), and N (non-M and non-O). M is the most predominant group of HIV-1 around the world [6]. Within the M group there are nine subtypes: A–D, F–H, J, and K. Among these, subtypes B are prevalent in most regions of the world such as USA, Europe, South East Asia, Australia, and South Africa [6].

Endogenous pathway of antigen processing and presentation is used to present endogenously synthesized cellular peptides as well as viral protein fragments via the MHC class I molecule to the cytotoxic-T-lymphocytes (CTLs). In this pathway, the proteins that are destined for the presentation are marked by the ubiquitinylation and subjected to proteolytic cleavage by the immunoproteasome. The fragments of peptides are transported to lumen of ER by the help of TAP (TAP1 and TAP2). These TAP proteins also help the loading of the short peptides with appropriate length (9 amino acids) into the groove of MHC class I molecule [7]. Although proteasome is the main player in generating the bulk of the CTL epitopes, cytosolic endopeptidases may also be involved in the production of certain CTL epitopes [8].

Peptides are typically tightly associated along their entire length in MHC class I groove. The N and C termini of the peptide are firmly H-bonded to the conserved residues of the MHC groove. The analysis of the naturally occurring peptides extracted from the MHC-peptide complex revealed that these are mostly 8-9 residues long and in certain key positions amino acids tend to be conserved within the peptide. These are called anchor positions which are proved to be essential for the binding of peptides to MHC class I molecules in allele specific manner. There are typically two (sometimes three) major anchor positions for the class I binding peptides. One is located at the C terminal end and the other one usually lies in the position 2 (P2) but also occurs in P3, 5, or 7 [7].

Cytotoxic-T-lymphocytes (CTLs) are one of the vital components of cellular immunity and play crucial role in eliminating viral infection. CTLs recognize viral antigen on the surface of virally infected cells in combination with appropriate major histocompatibility complex (MHC) molecule and exert their effect by killing the infected cells either by lysis or inducing apoptosis. Previous studies suggested that the HIV infection process can be divided into 3 stages. These are (1) acute viremia, (2) a latency period of variable time period, and (3) clinical AIDS. At the later stages of HIV infection CD4^+^ T cells counts drops down below 200 cells/mm^3^ which causes the complete collapse of immune response and consequently the opportunistic infectious agents such as* Pneumocystis carinii *come into the play [9]. There is now increasing body of evidence that CTLs play an important role in controlling the HIV infection. Analysis of the immune responses of the HIV infected patients revealed that antiviral CTL activity is correlated with clearance of virus particles during the acute phase of infection and a decline in the CTL activity is associated with the disease progression [9, 10]. Two types of antiviral CTL responses have been documented so far: one is classical viral epitope dependent-MHC restricted killing of virally infected cells and the other one is the noncytolytic response in which CTLs control the viral infection by inhibiting the viral replication [11]. Furthermore, studies of the SIV-macaque model, in which the administration of anti-CD8 monoclonal antibodies hinders the decline in viremia, provided strong evidence for the crucial role of CTLs in controlling HIV infection during acute phase [12]. Recently Goulder and Watkins have suggested three additional lines of evidence which signifies the potential role of CTLs in suppressing HIV infection: first they argued that specific HLA class I molecules are consistently associated with particular HIV disease outcomes. Secondly, they highlighted the fact that more rapid disease progression is observed in individuals with HLA class I homozygosity, and lastly they provided evidence that the loss of immune control over HIV infection arises when viral mutants escape CD8^+^ T-cell recognition [13]. All the above mentioned evidence signifies the important antagonizing role of CTL immune response in HIV disease progression.

## 2. Analysis of the HLA Class I Restricted CTL Epitopes in HIV Proteome

**Figure 1:**
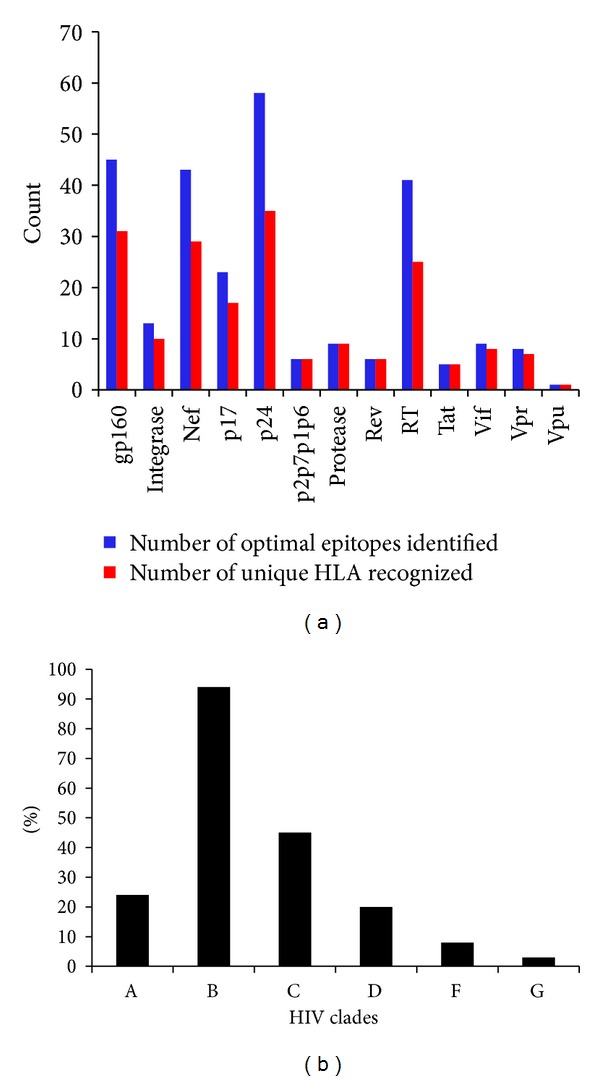
Number of optimal CTL epitopes and unique HLA recognized (a) and the percentage of clades (b) to which the optimal epitopes belong for whole HIV proteome were shown.

Design and development of HIV vaccine largely depend on our understanding of complex dynamics between host immune response and viral adaptation to selective pressure exerted by the host. Understanding how the CTL epitopes interact with particular HLA alleles can give an insight into the mechanisms of success or failure of immune control of a pathogen, such as HIV-1, for which clearance of virus particles depends on CTL activity. So the vaccine development strategies for HIV should be focused on identifying the epitopes presented by HLA alleles prevalent in populations severely affected by the global HIV epidemic. In recent years, development of new technologies such as measuring interferon-gamma (IFN*γ*) release by the enzyme linked immunospot (ELISPOT) assay and flow cytometry ensured the efficient evaluation of CTL responses against HIV epitopes [14]. Moreover, development of overlapping pooled peptide technology (OLP) now provides the opportunity for the detailed and precise analyses of HIV-1-specific cellular immune responses by elucidation of the T-cell epitopes and the identification of immunodominant regions of HIV-1 gene products. Identification and characterization of the CTL epitopes as well as the corresponding HLA alleles can play a major role in elucidating the nature of protective CTL response and mechanism of the immune evasion of HIV. A large number of HIV CTL epitopes have been identified and deposited into various databases. Apart from the experimental methods, various computational tools are now available which can predict CTL epitopes within viral proteome by using different sets of algorithms, for example, artificial neural network (ANN), average relative binding (ARB), stabilized matrix method (SMM), and so forth. The first CTL epitope was identified in 1988 by using synthetic peptide technology [15]. Since then, over 1200 HLA class I restricted HIV-1 epitopes were identified in HIV proteome (http://www.hiv.lanl.gov/content/immunology/index.html). In Table 2, a list of HLA class I allele restricted optimal CTL epitopes for HIV along with their corresponding HLA alleles and clades is given. For the identification of optimal epitopes, two criteria were imposed as described by Llanoa et al. [16]. These criteria include the unequivocal experimental validation of the epitope restriction by a specific HLA class I allele and the definition of the optimal epitope length (8 to 10 amino acid long). Analysis of the CTL epitopes listed in Table 2 reveals that epitopes from 5 HIV proteins (gp160, Nef, p24, p17, and RT) contributed 77% of the total epitopes listed in Table 2. The remaining percentage of the epitopes was derived from the eight other HIV proteins (Integrase, p2p7p1p6, Protease, Rev, Tat, Vpu, Vif and Vpr). The highest number of optimal epitopes was found for p24 (54) while the only one optimal epitope was identified for vpu (Figure 1). The epitope number for gp160, Nef, RT, and p17 were 45, 43, 41, and 23, respectively. The number of unique alleles recognized by these epitopes was also analyzed and found to be correlated with number of epitopes for each HIV protein (Figure 1). For instance, 54 p24 CTL epitopes were restricted cumulatively by 35 unique HLA class I alleles. Similarly, for the epitopes of gp160, Nef, p17, and RT, the numbers of unique HLA class I alleles were found to be 31, 29, and 25, respectively.

## 3. Clustering of CTL Epitopes in HIV Proteome

**Figure 2:**
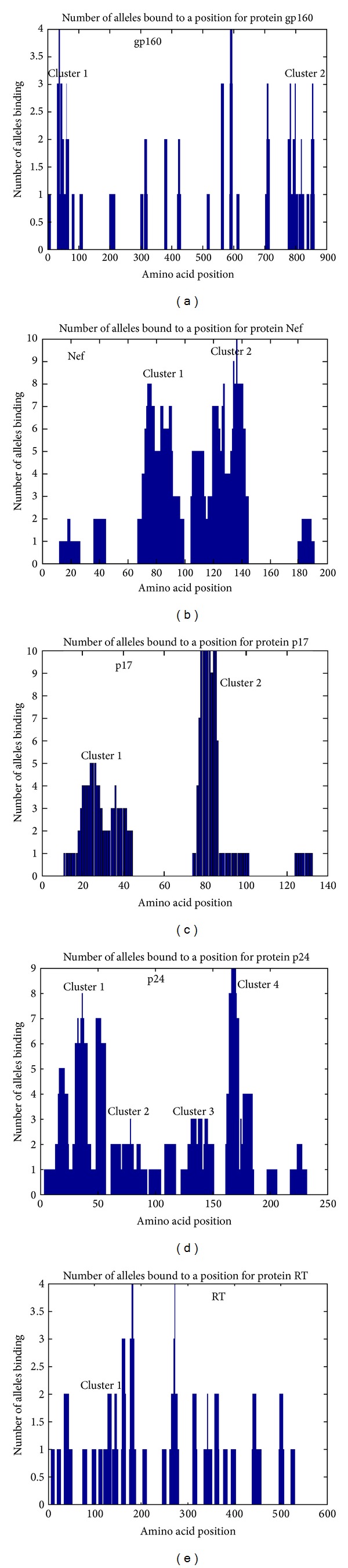
Clustering pattern of 5 HIV-1 proteins. The *X*-axis represents the amino acid position whereas the *Y*-axis represents the number of allele binding to particular positions.

Analysis of the HIV-1 proteins reveals that HLA class I restricted epitopes form overlapping clusters known as epitope rich/dense region whereas the regions deficient of any epitope clusters are called the epitope poor regions [17]. Yusim et al. have identified four short overlapping clusters in Nef protein of HIV-1 which was found to be multirestricted indicating that the clusters contain several epitopes recognized by different class I HLA molecules [18]. In another study, Currier et al. identified CTL epitope distribution patterns in the Gag and Nef proteins of HIV-1 from subtype-A infected subjects [19]. Studies aided with powerful experimental as well as computational methods are now being conducted with the aim to construct a fine CTL epitope map for the whole HIV-1 proteome. With the advancement of new sophisticated computational and statistical methods, it is now possible to identify the epitope clusters computationally. One significant achievement in computational immunology is the method of identification of immunoproteasome cleavage sites within the query proteins by using different algorithms such as artificial neural network (ANN) which enables the rapid identification of a wide range of potential epitopes that can be analyzed both computationally and experimentally for their affinity to bind with particular HLA class I molecules. Studies, dedicated to identify the CTL epitope clusters by means of computational methods, are now showing some success as far as the identification of new epitope clusters is concerned, as some novel epitope containing clusters were identified. However, more developments in the algorithms are required to construct more realistic models of epitope and cleavage site prediction, so that the predicted proteasomal cleavage events observed in calculation may better mimic the actual processing of viral antigens in the natural environment. In this study, the analysis of the topological arrangement of the 269 experimentally validated optimal epitopes in the HIV proteins listed in Table 2 allowed the identification of epitope clusters in the individual HIV proteins. Among the 13 different HIV proteins (listed in Table 2), epitope clustering was performed for 5 proteins (gp160, Nef, p17, p24, and RT) because for these proteins a relatively higher number of epitopes were identified (Figure 2 and Table 3). The aim of the cluster analysis is to identify the epitope dense regions or “hot spots” in the HIV-1 proteome. To cancel the possibility of random matching, the clusters containing more than 5 overlapping epitopes were only considered. For gp160, 2 major epitope clusters can be observed where the first (amino acid position: 31 to 69) and second (amino acid position: 770–838) cluster harboured 9 epitopes each. Like the gp160 protein 2 major clearly defined clusters were also identified for the Nef and p17 protein. In case of Nef, one spans from 68 to 100 and the second one lies between 105 and 145 amino acid positions. In previous study Penciolelli et al. [20] identified 4 clusters in the Nef protein which falls within the epitope cluster range for Nef observed in this study. For p17 protein, two clusters were similar in epitope composition and length. First p17 cluster with 34 amino acids was found to contain 10 epitopes whereas 2nd p17 cluster with 12 epitopes was composed of 30 amino acids. p24 was found to contain maximum numbers (4) of major epitope clusters. For RT only 1 major cluster was identified which contained 9 overlapping epitopes, whereas the rest of epitopes were found to be distributed randomly in protein.

Data from Table 3 suggest that epitopes in both the RT and gp160 proteins did not exhibit significant clustering properties compared to other HIV proteins. Only 40% and 22% of the epitopes were identified as part of the major cluster in gp160 and RT, respectively, which indicated that the majority of the epitopes were distributed randomly in respective proteins. Epitopes from other three proteins Nef, p17, and p24 showed significant clustering pattern as evident by both Figure 4 and Table 3. Most of the epitopes in these proteins were found to be a part of cluster or epitope dense region.

## 4. Are the CTL Epitope Clusters Conserved and Hydrophobic in Nature?

**Figure 3:**
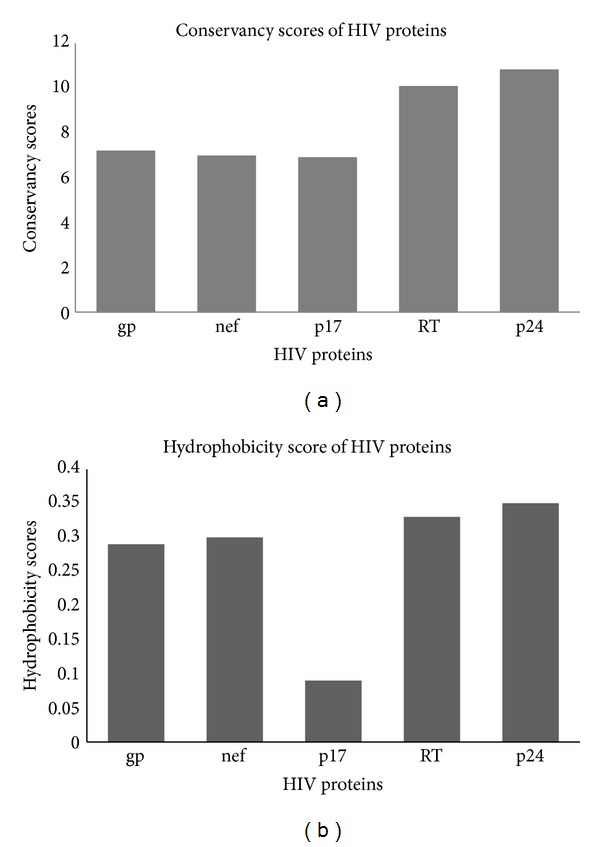
Cumulative conservancy (a) and hydrophobicity (b) scores of five individual proteins of HIV-1.

**Figure 4:**
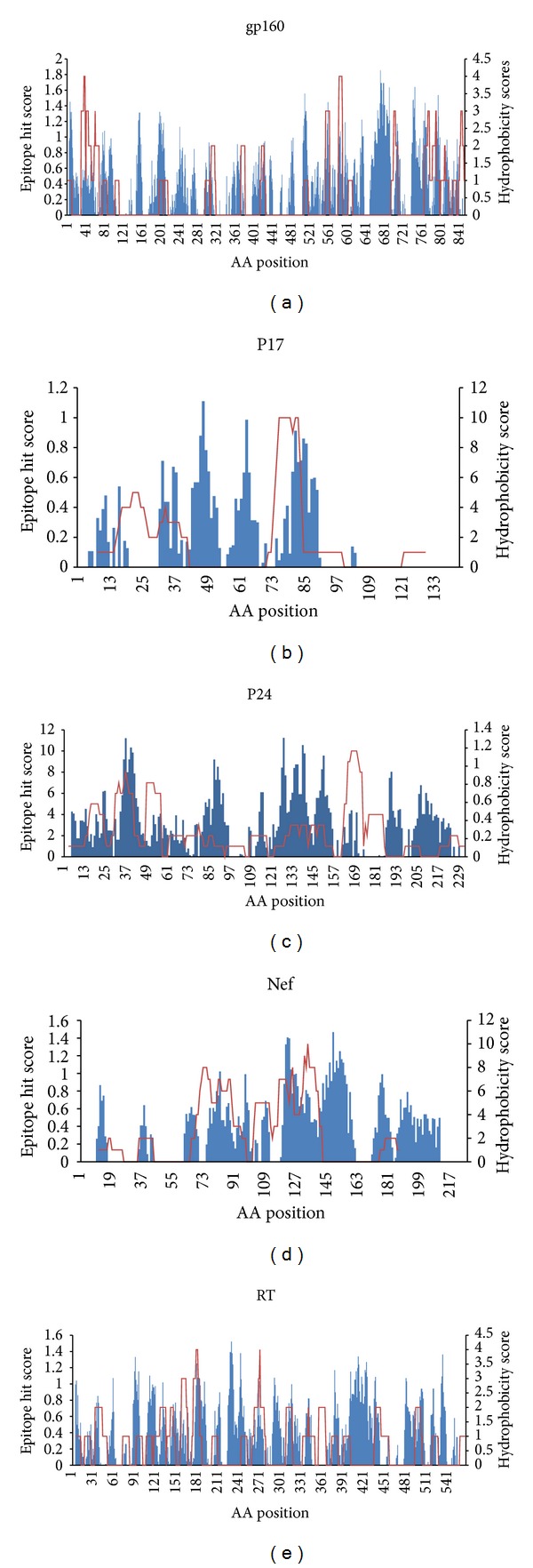
Epitope clusters and hydrophobic pattern of five HIV proteins. The *X*-axis represents the amino acid position. The primary *Y*-axis (left) shows the hydrophobicity scores whereas the secondary *Y*-axis (right) represents the epitope hit/count values. The blue bars indicate the hydrophobicity score whereas the red line represents the epitope hit values. For the hydrophobicity scores, the negative values were not shown in the figure, and only the scores greater than 0 were plotted.

By analyzing the nature of the CTL epitopes and their source proteins, Hughes and Hughes proposed two hypotheses about the nature of the CTL epitopes. First they proposed that the endogenous peptides presented by human leukocyte antigen (HLA) class I molecules are largely derived from conserved regions of proteins, so in general the CTL epitopes tend to be more conserved than the remainder portion of the source proteins. Secondly they hypothesized that the CTL epitope regions are hydrophobic whereas the source protein may itself be overall hydrophilic in nature [8]. In harmony with these hypotheses, Silva and Hughes showed that the CTL epitopes of HIV-1 Nef protein were derived from the hydrophobic and relatively conserved regions by estimating the relative conservation of CTL epitopes of the Nef protein and relating this to the structure and function of the protein. In another study Lucchiari-Hartz et al. showed that the CTL epitope clusters derived from Nef protein tend to coincide with hydrophobic regions, whereas the noncluster regions are predominantly hydrophilic [8]. Their* in vitro* analysis of the proteasomal degradation products of HIV-Nef protein demonstrates a differential sensitivity of cluster and noncluster regions to proteasomal processing and the cluster regions are digested by proteasomes with greater preference for hydrophobic P1 residues. But the authors admitted that some cytosolic endoproteases other than proteasomes may also be involved in the production of certain Nef-CTL epitopes in natural condition [8]. In both these studies the primary focus was on one protein (Nef) in the whole HIV proteome. So similar studies on other HIV proteins would certainly be interesting and could reveal some important feature of the HIV-CTL epitopes. In contrast to the notion that HIV CTL epitopes are more conserved and hydrophobic in nature, more recent study revealed that distribution of CTL epitopes in 99% of the HIV-1 protein sequences follows a random pattern and is indistinguishable from the distribution of CTL epitopes in proteins from other proteomes such as hepatitis C virus (HCV), influenza and for three eukaryote proteomes. In this study, the authors opted for the computational approach to predict the large set of CTL epitopes where proteasome cleavage pattern, TAP, and HLA-binding, three most crucial steps in classical endogenous antigen presentation pathway, were predicted by means of computational tools. The use of experimentally validated epitopes instead of computationally predicted epitopes could influence the outcome of the study and may lead the authors to a different conclusion. To shed some light on the contradiction of different studies mentioned above, an investigation involving hydrophobic and conservancy pattern of experimentally validated optimal CTL epitopes (Table 2) from 5 HIV proteins (gp160, Nef, p17, p24, and RT) was conducted in this study. Relative conservancy and hydrophobicity of the five selected HIV proteins were analyzed. 100 proteins sequences of different HIV clades retrieved from the Uniprot database (http://www.uniprot.org/) were used as an input for both conservancy and hydrophobic pattern prediction. To unveil the conservation pattern, multiple sequence alignment (MAS) was constructed using well stabled tool called Clustal W version 2.0 (http://www.ebi.ac.uk/Tools/clustalw2/index.html) developed by European Bioinformatics Institute (EBI). From the MSA the conservancy score for each amino acid position was obtained. To predict the hydrophobicity score Protscale tool of the ExPASy Proteomics Server (http://www.expasy.ch/tools/protscale-ref.html) and algorithm (developed by Abraham and Leo) previously used by Lucchiari-Hartz et al. [8] were employed. Both the hydrophobicity and conservancy scores for each amino acid position within a particular HIV protein were used to calculate the total scores for both these parameters.  Figure 3 shows the total hydrophobicity and conservancy scores of 5 individual HIV proteins in agreement with previous study [21]. We found that both the RT and p24 are relatively conserved and more hydrophobic than the rest of the analyzed HIV proteins (Figure 3). To visualize the overlapping pattern and correlation between the hydrophobic pattern and epitope clusters for the five selected proteins, the epitope count/hit and hydrophobicity scores were plotted together for each protein (Figure 4). The epitope hit score for a particular position is the number of alleles binding to that particular position.

To compare the correlation among hydrophobicity, conservancy, and epitope count, correlation coefficient was calculated among them (Appendix 1). For the calculation of correlation coefficient, first the standard deviation of each of the three score parameters (epitope count, hydrophobicity, and conservancy) was obtained (Appendix 1).  Table 4 shows the correlation score values among these three parameters.

From the correlation score it was evident that Nef epitope clusters were strongly correlated with the hydrophobicity and conservancy. p24 protein also showed relatively high correlation between epitope clusters and hydrophobicity and between conservancy and epitope clusters. In contrast, gp160 and RT showed relatively weak yet similar correlation among the parameters. p17 showed strong correlation when epitope hit was compared with conservancy but showed moderate correlation between epitope hit and hydrophobicity. So these findings suggested that not all the epitopes of HIV proteome are derived from conserved and hydrophobic regions of HIV-1 proteome although this hypothesis was found to be valid for two of the five HIV proteins (Nef and p24) as both of these proteins showed a significant correlation among epitope cluster, hydrophobicity, and conservancy. But the very weak correlation obtained for gp160 and RT diminished the general applicability of the hypothesis that all the HIV CTL epitopes were conserved and hydrophobic in nature.

## 5. The Role of MHC Class I on Immune Control of HIV Infection

Significant variation in the susceptibility to HIV-1 infection and especially in the clinical outcome after infection is observed in HIV infected patients. For instance, variation in the level of circulating virus particles in the plasma during the nonsymptomatic phase is commonly observed among the patients [22]. In addition to this, there is evidence that in certain cases individuals known as long-term nonprogressor (LTNP), infected with HIV, remain asymptomatic without antiretroviral therapy (ART) in their life time due to the slow or arrested evolution of HIV [23]. The most plausible explanation is that the variation in the susceptibility and outcome of HIV infection is largely due to host factors and viral adaptation to selective pressure. Recently Fellay et al. conducted a whole-genome association study to identify the host factor associated with control of HIV-1. In this study they identified two distinct polymorphisms associated with HLA loci B and C [24]. Surprisingly, almost all HLA class I polymorphisms were found to occur in those residues that belong to peptide-binding groove of these molecules thereby determining the epitopes that bind to each HLA molecule [25]. Among the three MHC class I loci in humans (HLA-A, HLA-B, and HLA-C), HLA-B is the most polymorphic, compared with HLA-A and HLA-C molecules (IMGT/HLA database: http://www.ebi.ac.uk/imgt/hla/). A more direct evidence of the association between HLA polymorphism and disease progression in HIV infected individual came from a previous study where they showed HLA-B*3503 associated with rapid disease progression differs in only one amino acid from HLA-B*3501 for which no such association was observed [26]. The presence of HLA-B*57 allele in a large proportion of LTNPs signifies its role in controlling disease progression and mutations in HLA-B*57-restricted Gag epitopes were frequently present in all viruses from plasma but interestingly inspite of this CTL escape mutations LTNPs can maintain viral suppression [27, 28]. The escape mutation in the HLA-B*57-restricted Gag epitopes can be considered as a consequence of strong evolutionary pressure exerted by the host immune response. Previous studies showed that although mutation in the conserved gag p24 epitope DRFYKTLRAE helps the virus to evade CTL response, it also impairs its ability to replicate because the mutation occurs in a very conserved position which is functionally constrained [29]. Among the three HLA class I molecules, HLA-B is considered as the most important factor for restricting HIV diseases progression and T cells responding to HLA-B-restricted epitopes appear to be immunodominant [30, 31]. Moreover, detailed study of the CTL epitopes in whole HIV-1 proteome revealed that HLA-B-restricted epitopes are more conserved compared to epitopes restricted by HLA-A and C. The same study also showed that although for most of the proteins the fractions of unique HLA-A and B restricted positions are equivalent in the total HIV clade-B proteome, Gag-p24 and Nef seemed to be preferentially targeted by HLA-B alleles as the B-restricted fractions were found to be over threefold higher than the A-restricted residues [32].

**Figure 5:**
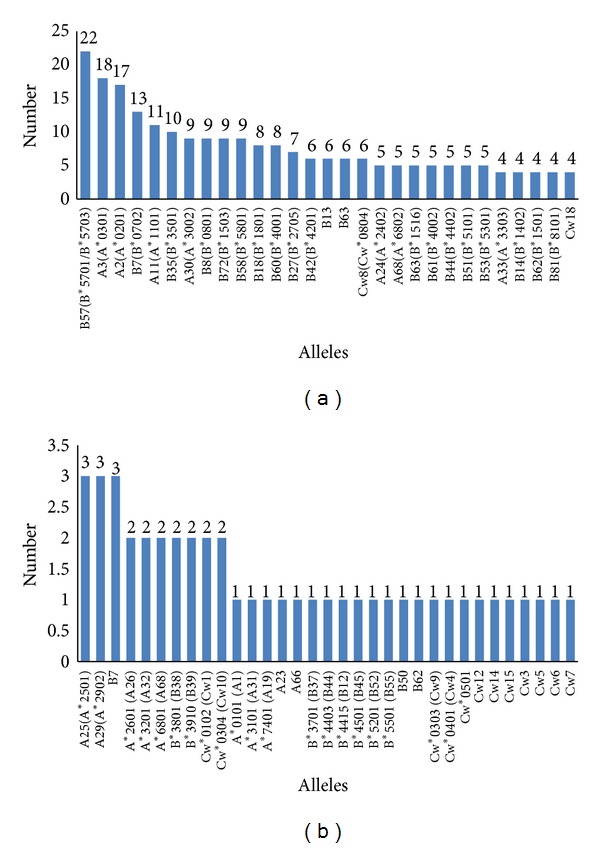
The number of unique alleles recognized by the optimal CTL epitopes.

In our study, the analysis of different class I HLA alleles that recognize all the listed CTL optimal epitopes revealed some interesting features of HLA restriction patterns of HIV-1 CTL epitopes.  Figure 5 shows the number of optimum epitopes recognized by 62 different class I HLA alleles. It was found that HLA-B*57 was the most successful as far as the number of epitope recognition was concerned as it recognizes 22 different optimal epitopes. The other successful HLA alleles were HLA-A*3, A*2, B*7, A*11, and B*35 (Figure 5). Among the total HLA alleles HLA-B contributes to the 50% of the total allele pool whereas HLA-A and C constitute 27% and 23%, respectively (Table 5).

These data also support the previous findings and also signify the role of HLA-B alleles in controlling HIV-1 infection as HLA-B was found to be associated with the 60% of the experimentally validated CTL epitopes. In harmony with the finding of Costaa et al. [32] we also observed a low % (9% of the total optimal epitope pool) of epitopes was recognized by HLA-C alleles. We have also analyzed the % of the epitopes associated with HLA-A and B alleles in individual HIV proteins (gp160, Nef, p24, p17, and RT). Analysis showed that only the p24 and Nef epitopes were associated with higher numbers of HLA-B alleles than A alleles. In contrast, epitopes derived from gp160, p17, protease, and other proteins (Tat, Rev, Vpu, Vpr, Vif) were recognized by slightly greater number of A alleles compared to B alleles. In case of intergrase, the epitopes were restricted by almost similar number of A and B alleles. There was a significant difference between the HLA*A and HLA*B associated epitopes for RT and p24. For RT the % of HLA*A and HLA*B associated epitopes are 19 and 6.28, respectively. In case of p24 the % of HLA*B associated epitopes are significantly higher (20%) than the HLA*A epitopes (3.5%).

## 6. Conclusion

As CTL response against HIV infected cells is proved to be crucial in controlling virus population in the host, rationally the CTL based vaccine should have a profound effect on HIV infection. Yusim et al. suggested that epitope clustering methods provide an alternative strategy to design novel multiepitope vaccine. They also suggested that the multiepitope vaccine should not be composed of a string of single epitopes, rather it should be composed of short region containing the epitope clusters and proximal regions flanking the epitope cluster that may be essential for optimal processing of epitopes. These epitope clusters harbor multiple overlapping epitopes which may be recognized by multiple HLA alleles. In conclusion, this study has shown the analysis of the HIV-1 CTL epitopes which revealed that Nef and p24 proteins of HIV-1 can be considered for CTL based multiepitope vaccine design since a significant number of optimal CTL epitopes are derived from Nef and Gag-p24 and almost all these epitopes showed a clustering pattern. A further study is needed to test these proposed vaccine candidates in laboratory animal to test safety and immunity against HIV.

## Figures and Tables

**Table 1:** Function of different HIV proteins.

Protein	Precursor	Functions
P17	Gag	Matrix protein p17 has two main functions. Firstly it targets Gag-pol polyproteins to the plasma membrane by the help of a membrane-binding signal which contains myristoylated N-terminus. Secondly it plays an essential role in the nuclear localization of the viral genome.

P24	Gag	Protein p24 forms the nucleocapsid that encapsulates the viral genomic RNA in the virion. The core is disassembled immediately after the entry of virion into host cell.

P7	Gag	Nucleocapsid protein p7 encapsulates viral genomic RNA and hence provides protection to viral genome. It binds these RNAs through its zinc finger motifs. It also acts as a nucleic acid chaperone as it tends to facilitate the rearrangement of nucleic acid secondary structure during reverse transcription of genomic RNA.

RT	Gag-pol	Reverse transcriptase/ribonuclease H (RT) is a multifunctional enzyme that facilitates the reverse transcription of viral RNA genome into dsDNA in the cytoplasm, shortly after virus entry into the cell. This enzyme also displays a DNA polymerase activity that can copy either DNA or RNA templates, and a ribonuclease H (RNase H) activity that cleaves the RNA strand of RNA-DNA heteroduplexes.

Integrase	Gag-pol	Integrase catalyzes integration of viral DNA into the host chromosome, by a multistep process involving DNA cutting and joining reactions.

Protease	Gag-pol	Cleavage of viral precursor polyproteins into mature proteins

Gp120	GP160	The surface protein gp120 (SU) facilitates the anchoring of the virus to the host target cell (CD4+) by binding to the primary receptor CD4. This interaction induces a change in the conformation exposing a high affinity binding site for a chemokine coreceptor (CXCR4 and/or CCR5) and promotes subsequent interaction between the envelope protein and CXCR4 and/or CCR5.

Tat		Tat acts as a nuclear transcriptional activator of viral gene expression that is essential for viral transcription from the LTR promoter. It also directs the components of the cellular transcription machinery into the viral RNA to promote transcription by the RNA polymerase complex.

Vif		It ensures the downregulation of APOBEC3G by recruiting the ubiquitin-proteasome machinery that targets APOBEC3G for degradation. It also binds to viral RNA and affects the stability of viral nucleoprotein core.

Vpr		It is largely involved in the transport of the viral preintegration (PIC) complex to the nucleus during the early phase of the infection. It probably interacts with karyopherin alpha/KPNA1 and KPNA2 thereby increasing their affinity for basic-type nuclear localization signal harboring proteins such as viral matrix protein, thus facilitating the translocation of the viral proteins into the nucleus.

Vpu		It promotes virion budding, by targeting human CD4 and CD317 to proteasomal degradation. CD4 degradation hinders any possible interactions between viral Env and human CD4 in the endoplasmic reticulum. It helps the proper Env assembly into virions.

Nef		(1) Downregulation of surface MHC-I molecules. (2) Downregulation of cell surface CD4 antigen. It interacts with the Src family kinase LCK and induces LCK-CD4 dissociation. Subsequently it causes clathrin-dependent endocytosis of CD4 antigen. Ultimately, the CD4 are decreased and infected cells. (3) It decreases the number of viral receptors and hence prevents reinfection by more HIV particles. (4) It prevents the apoptosis of the infected cell by inhibiting the Fas and TNFR-mediated death signals. It also interacts with p53 and protects the infected cell against p53-mediated apoptosis. Furthermore, it regulates the Bcl-2 family proteins through the formation of a Nef/PI3-kinase/PAK2 complex that induces phosphorylation of Bad.

**Table 2:** List of optimal CTL epitopes for HIV-1 (taken and modified from HIV molecular immunology database (http://www.hiv.lanl.gov/content/immunology/tables/optimal_ctl_summary.html).

HIV protein	AA position	HLA	Sequence	Clade
gp160	2–10	B*0801 (B8)	RVKEKYQHL	—
gp160	31–39	B*1801 (B18)	AENLWVTVY	B
gp160	31–39	B44	AENLWVTVY	B
gp160	31–40	B*4402 (B44)	AENLWVTVYY	—
gp160	37–46	A*0301 (A3)	TVYYGVPVWK	A, B, C, D
gp160	42–51	B*5501 (B55)	VPVWKEATTT	—
gp160	42–52	B*3501 (B35)	VPVWKEATTTL	B
gp160	52–61	A*2402 (A24)	LFCASDAKAY	—
gp160	59–69	B58	KAYETEVHNVW	C
gp160	61–69	B*1801 (B18)	YETEVHNVW	B
gp160	78–86	B*3501 (B35)	DPNPQEVVL	B
gp160	104–112	B*3801 (B38)	MHEDIISLW	B
gp160	199–207	A*1101 (A11)	SVITQACPK	B
gp160	209–217	A*2902 (A29)	SFEPIPIHY	B, D
gp160	298–307	B*0702 (B7)	RPNNNTRKSI	B, C
gp160	310–318	A*3002 (A30)	HIGPGRAFY	B
gp160	311–320	A*0201 (A2)	RGPGRAFVTI	A, B, C
gp160	375–383	B*1516 (B63)	SFNCGGEFF	A, B, C
gp160	375–383	Cw*0401 (Cw4)	SFNCGGEFF	A, B, C
gp160	416–424	B*5101 (B51)	LPCRIKQII	B
gp160	419–427	A*3201 (A32)	RIKQIINMW	B, C
gp160	511–519	Cw18	YRLGVGALI	C
gp160	557–565	Cw*0304 (Cw10)	RAIEAQQHL	A, B, C, D
gp160	557–565	Cw8	RAIEAQQHM	A, B, C, D
gp160	557–565	Cw15	RAIEAQQHL	C
gp160	584–592	B*1402 (B14)	ERYLKDQQL	A, B, C, D
gp160	585–593	A23	RYLKDQQLL	B, C
gp160	585–593	A*2402 (A24)	RYLKDQQLL	B, C
gp160	586–593	B*0801 (B8)	YLKDQQLL	A, B
gp160	606–614	B*3501 (B35)	TAVPWNASW	B
gp160	698–707	A*3303 (A33)	VFAVLSIVNR	B
gp160	703–712	A*2501 (A25)	EIIFDIRQAY	—
gp160	704–712	A*3002 (A30)	IVNRNRQGY	B
gp160	770–780	A*0301 (A3)	RLRDLLLIVTR	B, C
gp160	770–780	A*3101 (A31)	RLRDLLLIVTR	B, C
gp160	777–785	A*6802 (A68)	IVTRIVELL	B
gp160	786–795	B*2705 (B27)	GRRGWEALKY	B
gp160	787–795	A*0101 (A1)	RRGWEVLKY	B
gp160	794–802	A*3002 (A30)	KYCWNLLQY	B
gp160	805–814	B*4001 (B60)	QELKNSAVSL	B
gp160	813–822	A*0201 (A2)	SLLNATDIAV	B
gp160	831–838	A*3303 (A33)	EVAQRAYR	B
gp160	843–851	B*0702 (B7)	IPRRIRQGL	A, B, C, D
gp160	846–854	A*0205 (A2)	RIRQGLERA	B
gp160	848–856	B8	RQGLERALL	—
Integrase	28–36	B42	LPPIVAKEI	B, C
Integrase	66–74	B*1510 (B71)	THLEGKIIL	B, C
Integrase	123–132	B57	STTVKAACWW	B
Integrase	135–143	B*1503 (B72)	IQQEFGIPY	B, C
Integrase	165–172	Cw18	VRDQAEHL	C
Integrase	173–181	B*5701 (B57)	KTAVQMAVF	B
Integrase	179–188	A*0301 (A3)	AVFIHNFKRK	B, multiple
Integrase	179–188	A*1101 (A11)	AVFIHNFKRK	B, multiple
Integrase	185–194	B*1503 (B72)	FKRKGGIGGY	B, C
Integrase	203–211	A*1101 (A11)	IIATDIQTK	B
Integrase	219–227	A*3002 (A30)	KIQNFRVYY	AE, B, C, D
Integrase	260–268	B42	VPRRKAKII	—
Integrase	263–271	B*1503 (B72)	RKAKIIRDY	B, C
Nef	13–20	B*0801 (B8)	WPTVRERM	B
Nef	19–27	B62	RMRRAEPAA	B
Nef	37–45	B*4001 (B60)	LEKHGAITS	B
Nef	37–45	B50	LEKHGAITS	B
Nef	68–76	B*0702 (B7)	FPVTPQVPL	B
Nef	68–77	B*0702 (B7)	FPVTPQVPLR	B
Nef	71–79	B*0702 (B7)	TPQVPLRPM	B
Nef	71–79	B*4201 (B42)	RPQVPLRPM	B, C
Nef	73–82	A*0301 (A3)	QVPLRPMTYK	A, B, C, D
Nef	73–82	A*1101 (A11)	QVPLRPMTYK	A, B, C, D
Nef	74–81	B*3501 (B35)	VPLRPMTY	A, B, C, D
Nef	75–82	A*1101 (A11)	PLRPMTYK	B
Nef	77–85	B*0702 (B7)	RPMTYKAAL	B
Nef	82–91	Cw8	KAAVDLSHFL	B
Nef	83–91	A*0205 (A2)	GAFDLSFFL	A
Nef	83–91	Cw3	AALDLSHFL	B
Nef	83–91	Cw*0802 (Cw8)	AAVDLSHFL	B, C
Nef	84–92	A*0301 (A3)	AVDLSHFLK	A, B, D, F
Nef	84–92	A*1101 (A11)	AVDLSHFLK	A, B, D, F
Nef	90–97	B*0801 (B8)	FLKEKGGL	A, B, C, D
Nef	92–100	B*4001 (B60)	KEKGGLEGL	B, C
Nef	92–100	B*4002 (B61)	KEKGGLEGL	B, C
Nef	105–114	B*2705 (B27)	RRQDILDLWI	B
Nef	105–115	B18	RRQDILDLWVY	B
Nef	105–115	Cw7	KRQEILDLWVY	B, C
Nef	106–114	B13	RQDILDLWI	B
Nef	106–115	B*0702 (B7)	RQDILDLWIY	—
Nef	116–124	B57	HTQGYFPDW	B, C
Nef	116–125	B*5701 (B57)	HTQGYFPDWQ	B, C
Nef	117–127	B*1501 (B62)	TQGYFPDWQNY	B, C
Nef	120–128	A29	YFPDWQNYT	B, C
Nef	120–128	B*3701 (B37)	YFPDWQNYT	B, C
Nef	120–128	B*5701 (B57)	YFPDWQNYT	B, C
Nef	120–128	Cw6	YFPDWQNYT	B, C
Nef	127–135	B57	YTPGPGIRY	B, C
Nef	127–135	B63	YTPGPGIRY	B, C
Nef	128–137	B*0702 (B7)	TPGPGVRYPL	B, C
Nef	128–137	B*4201 (B42)	TPGPGVRYPL	B, C
Nef	133–141	A33	TRYPLTFGW	B
Nef	134–141	A*2402 (A24)	RYPLTFGW	B, C
Nef	135–143	B*1801 (B18)	YPLTFGWCY	B, C, D
Nef	135–143	B53	YPLTFGWCF	B
Nef	135–143	B*5301 (B53)	YPLTFGWCY	B
Nef	136–145	A*0201 (A2)	PLTFGWCYKL	B
Nef	137–145	B57	LTFGWCFKL	A, B, C
Nef	137–145	B63	LTFGWCFKL	A, B, C
Nef	180–189	A*0201 (A2)	VLEWRFDSRL	B
Nef	183–191	B*1503 (B72)	WRFDSRLAF	B
p17	11–19	B*4002 (B61)	GELDRWEKI	B
p17	18–26	A*0301 (A3)	KIRLRPGGK	A, B
p17	19–27	B*2705 (B27)	IRLRPGGKK	B
p17	20–28	A*0301 (A3)	RLRPGGKKK	A, B
p17	20–29	A*0301 (A3)	RLRPGGKKKY	B
p17	24–32	B*0801 (B8)	GGKKKYKLK	B, F
p17	28–36	A*2402 (A24)	KYKLKHIVW	B, C, F
p17	33–41	Cw*0804 (Cw8)	HLVWASREL	C
p17	34–44	A30	LVWASRELERF	B, C
p17	36–44	B*3501 (B35)	WASRELERF	B, C
p17	74–82	B*0801 (B8)	ELRSLYNTV	F
p17	76–86	A*3002 (A30)	RSLYNTVATLY	B, C, F
p17	76–86	B58	RSLYNTVATLY	B, C, F
p17	76–86	B63	RSLYNTVATLY	B, C, F
p17	77–85	A*0201 (A2)	SLYNTVATL	A, B, C, D, F, G, K
p17	77–85	A*0202 (A2)	SLYNTVATL	A, B, C, D, F, G, K
p17	77–85	A*0205 (A2)	SLYNTVATL	A, B, C, D, F, G, K
p17	78–85	Cw14	LYNTVATL	B, D
p17	78–86	A*2902 (A29)	LYNTVATLY	B, C
p17	78–86	B*4403 (B44)	LYNTVATLY	B, C
p17	84–91	A*1101 (A11)	TLYCVHQK	—
p17	92–101	B*4001 (B60)	IEIKDTKEAL	B, F
p17	124–132	B*3501 (B35)	NSSKVSQNY	B
p24	3–11	B13	VQNLQGQMV	B, C
p24	12–20	B*1510 (B71)	HQAISPRTL	B
p24	13–23	A*2501 (A25)	QAISPRTLNAW	B
p24	15–23	B*5701 (B57)	ISPRTLNAW	A, C
p24	15–23	B63	ISPRTLNAW	A, B, C, D
p24	16–24	B*0702 (B7)	SPRTLNAWV	B
p24	24–32	B*1503 (B72)	VKVIEEKAF	B, C
p24	28–36	B*4415 (B12)	EEKAFSPEV	A, B, C, D
p24	30–37	B*5703 (B57)	KAFSPEVI	B
p24	30–40	B*5701 (B57)	KAFSPEVIPMF	A, B, C, G
p24	30–40	B*5703 (B57)	KAFSPEVIPMF	A, B, C, G
p24	30–40	B63	KAFSPEVIPMF	A, B, C, G
p24	32–40	B57	FSPEVIPMF	B, C
p24	35–43	A*2601 (A26)	EVIPMFSAL	A, B, C, D
p24	36–43	Cw*0102 (Cw1)	VIPMFSAL	B, D
p24	44–52	B*4001 (B60)	SEGATPQDL	B
p24	48–56	B*0702 (B7)	TPQDLNTML	A, B, C, D
p24	48–56	B*3910 (B39)	TPQDLNTML	A, B, C, D
p24	48–56	B*4201 (B42)	TPQDLNTML	A, B, C, D
p24	48–56	B*5301 (B53)	TPYDINQML	A
p24	48–56	B*8101 (B81)	TPQDLNTML	A, B, C, D
p24	48–56	Cw*0802 (Cw8)	TPQDLNTML	A, B, C, D
p24	61–69	B*1510 (B71)	GHQAAMQML	B, C
p24	61–69	B*3901 (B39)	GHQAAMQML	B, C
p24	70–78	B*4002 (B61)	KETINEEAA	B
p24	71–80	A*2501 (A25)	ETINEEAAEW	A, B, D
p24	78–86	B*4002 (B61)	AEWDRVHPV	B
p24	84–92	B7	HPVHAGPIA	B, C, D, F
p24	94–104	B13	GQMREPRGSDI	B, C
p24	108–117	B*5701 (B57)	TSTLQEQIGW	B, C
p24	108–117	B*5801 (B58)	TSTLQEQIGW	B, C
p24	122–130	B*3501 (B35)	PPIPVGDIY	A, B, C
p24	128–135	B*0801 (B8)	EIYKRWII	B
p24	131–140	B*2703 (B27)	RRWIQLGLQK	—
p24	131–140	B*2705 (B27)	KRWIILGLNK	A, B, C, D
p24	137–145	B*1501 (B62)	GLNKIVRMY	A, B
p24	142–150	Cw18	VRMYSPVSI	B, C, F
p24	143–150	B*5201 (B52)	RMYSPTSI	B, F
p24	161–169	Cw18	FRDYVDRFF	C
p24	161–170	B*1801 (B18)	FRDYVDRFYK	B, D
p24	162–172	A*2402 (A24)	RDYVDRFFKTL	A
p24	162–172	B*4402 (B44)	RDYVDRFYKTL	B, D
p24	164–172	Cw*0303 (Cw9)	YVDRFFKTL	A, C, D
p24	164–172	A*0207 (A2)	YVDRFYKTL	B
p24	164–172	B*1503 (B72)	YVDRFFKTL	A, C, D
p24	164–172	Cw*0304 (Cw10)	YVDRFFKTL	A, C, D
p24	166–174	B*1402 (B14)	DRFYKTLRA	B, D
p24	174–184	B*4402 (B44)	AEQASQDVKNW	B, C, D
p24	174–185	Cw5	AEQASQEVKNWM	—
p24	176–184	B*5301 (B53)	QASQEVKNW	B, D
p24	176–184	B*5701 (B57)	QASQEVKNW	C
p24	197–205	B*0801 (B8)	DCKTILKAL	B
p24	217–227	A*1101 (A11)	ACQGVGGPGHK	B
p24	223–231	B*0702 (B7)	GPGHKARVL	B, C, D, F
Protease	3–11	A*6802 (A68)	ITLWQRPLV	A, B, C, D
Protease	3–11	A*7401 (A19)	ITLWQRPLV	A, B, C, D
Protease	30–38	A*6802 (A68)	DTVLEEWNL	D
Protease	34–42	B44	EEMNLPGRW	B
Protease	57–66	B13	RQYDQILIEI	B
Protease	68–76	B*1503 (B72)	GKKAIGTVL	BC
Protease	70–77	B57	KAIGTVLV	BC
Protease	76–84	A*0201 (A2)	LVGPTPVNI	B
Protease	80–90	B81	TPVNIIGRNML	C
Rev	14–23	B*5701 (B57)	KAVRLIKFLY	B
Rev	14–23	B*5801 (B58)	KAVRLIKFLY	B
Rev	14–23	B63	KAVRLIKFLY	B
Rev	41–50	B7	RPAEPVPLQL	A, B, C, D, F
Rev	57–66	A*0301 (A3)	ERILSTYLGR	B
Rev	67–75	Cw*0501	SAEPVPLQL	B
RT	5–12	B*4001 (B60)	IETVPVKL	B
RT	18–26	B*0801 (B8)	GPKVKQWPL	A, B, C, D
RT	33–41	A*0201 (A2)	ALVEICTEM	B
RT	33–43	A*0301 (A3)	ALVEICTEMEK	B
RT	42–50	B*5101 (B51)	EKEGKISKI	B
RT	73–82	A*0301 (A3)	KLVDFRELNK	B
RT	93–101	A*0301 (A3)	GIPHPAGLK	B
RT	107–115	B*3501 (B35)	TVLDVGDAY	AG, B
RT	118–127	B*3501 (B35)	VPLDEDFRKY	B, C
RT	127–135	A2	YTAFTIPSV	—
RT	128–135	B*5101 (B51)	TAFTIPSI	B
RT	137–146	B18	NETPGIRYQY	B, C
RT	142–149	B*1401 (B14)	IRYQYNVL	C
RT	156–164	B7	SPAIFQSSM	A, B, C, D
RT	158–166	A*0301 (A3)	AIFQSSMTK	A, B, C, D
RT	158–166	A*1101 (A11)	AIFQSSMTK	—
RT	173–181	A*3002 (A30)	KQNPDIVIY	B
RT	175–183	B18	NPEIVIYQY	C
RT	175–183	B*3501 (B35)	HPDIVIYQY	A, B
RT	179–187	A*0201 (A2)	VIYQYMDDL	A, B, C, D
RT	202–210	B*4001 (B60)	IEELRQHLL	B
RT	244–252	B*5701 (B57)	IVLPEKDSW	B
RT	260–271	B*1501 (B62)	LVGKLNWASQIY	B
RT	263–271	A*3002 (A30)	KLNWASQIY	B, C
RT	269–277	A*0301 (A3)	QIYPGIKVR	B, C
RT	271–279	B*4201 (B42)	YPGIKVRQL	B, C
RT	309–317	A*0201 (A2)	ILKEPVHGV	A, B, C, D
RT	309–318	B*1501 (B62)	ILKEPVHGVY	A, B, D
RT	333–341	B13	GQGQWTYQI	B
RT	341–350	A*1101 (A11)	IYQEPFKNLK	B, C
RT	356–365	A*3002 (A30)	RMRGAHTNDV	B
RT	356–366	A*0301 (A3)	RMRGAHTNDVK	B
RT	375–383	B*5801 (B58)	IAMESIVIW	B, C
RT	392–401	A*3201 (A32)	PIQKETWETW	B
RT	436–445	A*6802 (A68)	GAETFYVDGA	B, C
RT	438–448	A66	ETFYVDGAANR	B, C
RT	449–457	A*2601 (A26)	ETKLGKAGY	B
RT	495–503	Cw*0802 (Cw8)	IVTDSQYAL	—
RT	496–505	B*1503 (B72)	VTDSQYALGI	—
RT	520–528	A*1101 (A11)	QIIEQLIKK	B
RT	560–568	B81	LFLDGIDKA	—
Tat	2–11	B*5301 (B53)	EPVDPRLEPW	B
Tat	2–11	B58	EPVDPRLEPW	B
Tat	30–37	Cw12	CCFHCQVC	B
Tat	38–47	B*1503 (B72)	FQTKGLGISY	C
Tat	39–49	A*6801 (A68)	ITKGLGISYGR	B
Vif	17–26	A*0301 (A3)	RIRTWKSLVK	B
Vif	28–36	A*0301 (A3)	HMYISKKAK	B
Vif	31–39	B*5701 (B57)	ISKKAKGWF	B
Vif	48–57	B*0702 (B7)	HPRVSSEVHI	B
Vif	57–66	B51	IPLGDAKLII	B
Vif	79–87	B*1510 (B71)	WHLGHVSI	B
Vif	79–87	B*3801 (B38)	WHLGQGVSI	B
Vif	102–111	B*1801 (B18)	LADQLIHLHY	B
Vif	158–168	A*0301 (A3)	KTKPPLPSVKK	B
Vpr	29–37	B51	EAVRHFPRI	B
Vpr	30–38	B*5701 (B57)	AVRHFPRIW	B, C
Vpr	31–39	B27	VRHFPRIWL	B
Vpr	34–42	B*0702 (B7)	FPRIWLHGL	B
Vpr	34–42	B*8101 (B81)	FPRIWLHGL	B
Vpr	48–57	A*6802 (A68)	ETYGDTWTGV	C
Vpr	52–62	A*6801 (A68)	DTWAGVEAIIR	B
Vpr	59–67	A*0201 (A2)	AIIRILQQL	B
Vpu	29–37	A*3303 (A33)	EYRKILRQR	B
Gag-Pol	24–31	Cw*0102 (Cw1)	NSPTRREL	—
p2p7p1p6	1–10	B*4501 (B45)	AEAMSQVTNS	—
p2p7p1p6	42–50	B14	CRAPRKKGC	B
p2p7p1p6	64–71	B*4002 (B61)	TERQANFL	B
p2p7p1p6	66–74	B13	RQANFLGKI	B, C
p2p7p1p6	70–79	A*0201 (A2)	FLGKIWPSYK	B
p2p7p1p6	118–126	B*4001 (B60)	KELYPLTSL	B

**Table 3:** Analysis of the identified epitope clusters in HIV-1 proteins.

HIV-1 protein	Total epitope number	Cluster number	Cluster amino acid position	Cluster length (amino acids)	Number of epitopes in cluster	% of clustered epitopes in total epitope pool
Gp160	45	1	31–69	39	9	40
		2	770–838	69	9	

Nef	43	1	68–100	33	18	97
		2	105–145	41	24	

p17	23	1	11–44	34	10	95
		2	72–101	30	12	

p24	54	1	3–56	54	22	98
		2	61–117	57	9	
		3	128–150	43	9	
		4	161–184	24	13	

RT	41	1	107–166	60	9	22

**Table 4:** Correlation coefficient.

Correlation score	HIV-1 proteins				
	gp160	Nef	p17	p24	RT
Epitope hit versus hydrophobicity	0.11	0.27	0.16	0.26	0.12
Epitope hit versus conservancy	0.097	0.21	0.19	0.23	0.047
Hydrophobicity versus conservancy	0.20	0.22	0.23	0.15	0.009

**Table 5:** Analysis of the different HLA alleles.

HLA	Total number and as % of total HLAs	Number of epitope recognized	% of epitope recognized by HLA in total epitope pool
A	17 (27%)	86	31%
B	31 (50%)	165	60%
C	14 (23%)	24	9%

## Appendix

*Correlation between Different Scores.* There are three scores for each of the *k*-th position of epitope:

- epitope hit score: EHS(*k*),
- hydrophobicity score: HS(*k*),
- conservancy score: CS(*k*).

Let us assume that the position range of epitope, for which each of scores exists, is *j* to *n*.

Now

- the average epitope hit score EHS_AVG = (1/(*n* − *j* + 1))∑_*k*=*j*_^*n*^EHS(*k*);
- the average hydrophobicity score HS_AVG = (1/(*n* − *j* + 1))∑_*k*=*j*_^*n*^HS(*k*);
- the average conservancy score CS_AVG = (1/(*n* − *j* + 1))∑_*k*=*j*_^*n*^CS(*k*).

Now

(a) the standard deviation of epitope hit score
  (A.1) $$\begin{array}{c} \text{EHS}\_\text{SD}=\frac{1}{n-j+1}\overset{n}{\underset{k=j}{\sum }}{\left(\text{EHS}\left(k\right)-\text{EHS\_AVG}\right)}^{2}. \end{array}$$
(b) The standard deviation of hydrophobicity score
  (A.2) $$\begin{array}{c} \text{HS}\_\text{SD}=\frac{1}{n-j+1}\overset{n}{\underset{k=j}{\sum }}{\left(\text{HS}\left(k\right)-\text{HS\_AVG}\right)}^{2}. \end{array}$$
(c) The standard deviation of conservancy score
  (A.3) $$\begin{array}{c} \text{CS}\_\text{SD}=\frac{1}{n-j+1}\overset{n}{\underset{k=j}{\sum }}{\left(\text{CS}\left(k\right)-\text{CS\_AVG}\right)}^{2}. \end{array}$$

Then the correlation coefficient between the scores are calculated as below.

Correlation between epitope hit count and hydrophobicity score

(A.4) Corr (EHS, HS)  =∑k=jn(EHS(k)−EHS_AVG)(HS(k)−HS_AVG)(n−j)EHS_SD×HS_SD.

Correlation between epitope hit count and conservancy score

(A.5) Corr (EHS, CS)  =∑k=jn(EHS(k)−EHS_AVG)(CS(k)−CS_AVG)(n−j)EHS_SD×CS_SD.

Correlation between conservancy and hydrophobicity score

(A.6) Corr (CS, HS)  =∑k=jn(CS(k)−CS_AVG)(HS(k)−HS_AVG)(n−j)CS_SD×HS_SD.

The calculation of each of these correlations is carried out for each protein group separately.
